# Epigenetic programming of Dnmt3a mediated by AP2*α* is required for granting preadipocyte the ability to differentiate

**DOI:** 10.1038/cddis.2016.378

**Published:** 2016-12-01

**Authors:** Wei Guo, Jiangnan Chen, Ying Yang, Jianbei Zhu, Jiarui Wu

**Affiliations:** 1 Key Laboratory of Systems Biology, Institute of Biochemistry and Cell Biology, Shanghai Institutes for Biological Sciences, Chinese Academy of Sciences, Shanghai, China; 2 School of Life Science and Technology, ShanghaiTech University, Shanghai, China; 3 School of Life Science, University of Chinese Academy of Sciences, Shanghai,China; 4 Shanghai Advanced Research Institute, Chinese Academy of Sciences, Shanghai, China

## Abstract

Adipogenesis has an important role in regulating energy homeostasis in mammals. 3T3-L1 preadipocytes have been widely used as an *in vitro* model for analyzing the molecular mechanism of adipogenesis. Previous reports indicated that the stage of contact inhibition (CI), through which the proliferating cells exit from the cell cycle, was required for granting preadipocyte the ability to differentiate. While this kind of the granting mechanism remains elusive. In the present study, we showed that DNA (cytosine-5) methyltransferase 3a (Dnmt3a) was upregulated at both the mRNA and protein level during the CI stage, and resulted in increasing promoter methylation of adipogenic genes. We further identified that the expression of Activator protein 2*α* (AP2*α*), a member of the transcription factor activator protein 2 (AP2) family, was highly correlated with the expression of Dnmt3a during the CI stage. In addition, we showed that AP2*α* transcriptionally upregulated Dnmt3a by directly binding to its proximal promoter region. Importantly, treatment of 3T3-L1 preadipocytes with AP2*α*-specific siRNAs inhibited the preadipocyte differentiation in a stage-dependent manner, supporting the conclusion that AP2*α* has an important role during the CI stage. Furthermore, overexpression of Dnmt3a partially rescued the impairment of adipogenesis induced by AP2*α* knockdown. Collectively, our findings reveal that AP2*α* is an essential regulator for granting preadipocyte the ability to differentiate through the upregulation of Dnmt3a expression during the CI stage.

Adipogenesis has an important role in energy homeostasis of mammals. Better understanding the mechanism underlying adipogenesis may provide novel therapeutic targets in the treatment of obesity and other metabolic diseases.^1, 2, 3, 4^ 3T3-L1 preadipocytes have been widely used as an *in vitro* model for studying the molecular mechanisms governing adipogenesis.^5, 6^ 3T3-L1 preadipocytes first undergo growth arrest by contact inhibition (CI), and then differentiate into adipocytes upon exposure to a hormone cocktail including isobutylmethylxanthine, dexamethasone and insulin (MDI). A number of reports have revealed that the MDI-induced differentiation is controlled by a complicated network of transcription factors, in which CCAT-enhancer binding proteins (C/EBPs) and peroxisome proliferator-activated receptor-*γ* (PPAR*γ*) have critical roles.^7, 8, 9, 10, 11^ Although some previous studies reported a few factors such as transcriptional factor 7-like 1, GATA-2 and GATA-3 that were involved in adipogenic competency during the CI stage,^12, 13, 14^ the factors that function for regulation of adipogenesis in the CI stage are largely unknown.

Our previous study showed that the differentiation process of 3T3-L1 cells could be subdivided into licensing and execution phases.^15, 16^ In the licensing phase that is about 48 h of the cell–cell CI, proliferating preadipocytes exit from the cell cycle and are granted the ability to differentiate. Upon MDI stimulation, those licensed preadipocytes enter the execution phase for terminal differentiation. Importantly, our previous data showed that epigenetic modification such as DNA methylation in the licensing stage was involved in granting preadipocytes the ability to differentiate.^15^ We first showed that adipogenesis of 3T3-L1 cells treated by DNA methylation inhibitors (5-aza-2'-deoxycytidine (5-aza-dC) or 5-azacytidine (5-aza)) during the CI stage was markedly suppressed, whereas treatment of preadipocytes at the executive stage was less sensitive to these DNA methylation inhibitors.^15^ In addition, we found that DNA (cytosine-5) methyltransferase 3a (Dnmt3a) was involved in this epigenetic programming during the CI stage.^15^ Disturbing Dnmt3a with siRNA during the CI stage leads to significant reduction of adipogenesis. More efforts are required to provide mechanistic insights into the epigenetic programming during the CI stage.

Activator protein 2*α* (AP2*α*) is a sequence-specific DNA binding transcription factor. Its homo- and heterodimers could transactivate target genes by binding to GC-rich consensus elements.^17^ AP2*α* has an important role as the regulator during vertebrate development, cell growth and differentiation, carcinogenesis.^18, 19, 20, 21^ In 1998, Jiang *et al.*^22^ found that the expression of AP2*α* was markedly elevated during the CI stage in preadipocytes and declined rapidly after MDI induction, and they further showed that AP2*α* was responsible for delaying C/EBP*α* expression to ensure the progression of mitotic clonal expansion in adipogenesis.^23, 24, 25^ Recently, another study showed that epigenetic histone modifications underlay AP2*α*-mediated inhibition of C/EBP*α* expression.^26^ However, the role of the elevation of AP2*α* during the CI stage in adipogenesis remains elusive.

In the present study, we showed that AP2*α* during the CI stage was required for granting 3T3-L1 preadipocytes the ability to differentiate through upregulating Dnmt3a expression. In addition, AP2*α* was proved to be a transactivator of *Dnmt3a* gene by directly binding to its proximal promoter. AP2*α* knockdown during the CI stage impaired both DNA methylation and adipogenesis greatly, and then the overexpression of Dnmt3a was able to rescue the impaired adipogenesis caused by AP2*α* knockdown. Our findings deliver an AP2*α*-mediated epigenetic programming for granting preadipocytes the ability to differentiate in the licensing stage.

## Results

### Upregulation of Dnmt3a is associated with increasing promoter methylation of key adipogenic genes during the CI stage

**Figure 1:**
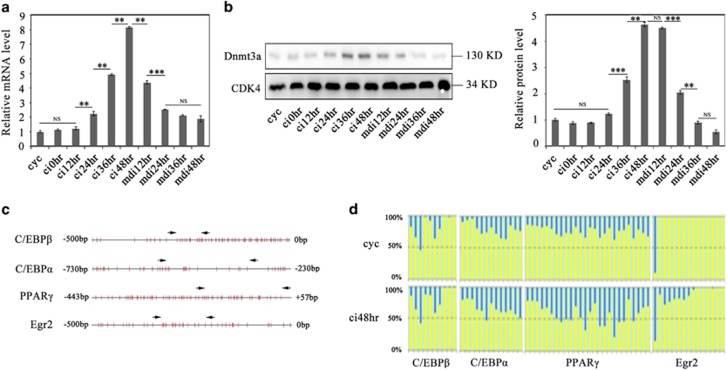
Dnmt3a is upregulated by AP2*α* during the CI stage. (**a**) Illustration of the murine *Dnmt3a* promoter (−1.7 to +0.1 kb) is shown and two reported AP2*α* binding sites were identified in the proximal region. (**b**) Correlation coefficient analysis of AP2*α* and Dnmt3a mRNA expression profiles during the CI stage. The mRNA expressions of AP2*α* and Dnmt3a at different time points during the CI stage were determined by real-time PCR and normalized to glyceraldehyde-3-phosphate dehydrogenase. (**c**) Real-time PCR analysis of the expression of Dnmt3a in AP2*α* knockdown cells (si-1 and si-2) at ci48 h. The results were analyzed as means±S.D. (*n*=3). Statistical significance is indicated: ***P<0.001. (**d**) Western blotting analysis of the expression of Dnmt3a in AP2*α* knockdown cells at ci48 h. Cyclin-dependent kinase 4 served as the loading control. Quantification analysis of the relative protein level of Dnmt3a in AP2*α* knockdown cells was shown on the right panel. The results were analyzed as means±S.D. (*n*=3). Statistical significance is indicated: **P<0.01. (**e**) A luciferase reporter plasmid of the murine *Dnmt3a* promoter (−1.7 to +0.1 kb) and an HA-AP2*α* cDNA plasmid were constructed for dual-luciferase assay. Results were expressed as firefly luciferase activity normalized to renilla luciferase activity. Error bars indicate S.D. (*n*=3). Statistical significance is indicated: *P<0.05, **P<0.01. Gradient expression of HA-AP2*α* was detected by western blotting (bottom panel)

Our previous study showed that administration with DNA methyltransferase inhibitors or Dnmt3a-specific siRNA at the CI stage reduced adipogenesis more significantly than that after the CI stage (Figures 2c and d in Guo *et al.*^15^) implying that epigenetic programming during the CI stage is required for adipogenesis. To further understand this observation, we first examined the expression profile of Dnmt3a from cycling cells to the MDI-induced cells. Quantitative real-time PCR (qRT-PCR) and western blot analysis showed that the expression levels of both mRNA and protein of Dnmt3a in preadipocytes increased gradually during the CI stage and then decreased significantly after 24 h of the MDI induction (Figures 1a and b), indicating that Dnmt3a is upregulated specifically in the CI stage.

Our previous study revealed that the methylation level of the *C/EBPα* promoter region during the CI stage is higher than that in the MDI-induced stage (Figure 4b in Guo *et al.*^15^). In the present study, we further measured the alteration in the promoter methylation of 9 important adipogenic genes (Supplementary Figure 1a) from the cycling stage to the CI stage with bisulfite sequencing. The results showed that the promoter methylation of murine TFs *C/EBPβ*, *C/EBPα*, *PPARγ* and early growth response 2 *(Egr2*) significantly increased during the CI stage (Figure 1d), whereas the promoter methylation of other five adipogenic genes has no obvious change (data not shown). The illustration of the 5' flanking and 5'untranslated sequences of *C/EBPβ*, *C/EBPα*, *PPARγ* and *Egr2* is shown and the red vertical line stands for CpG sites, and the regions indicated by arrows stand for the sequence for bisulfite sequencing (Figure 1c). In addition, we found later that the knockdown of AP2*α*, a transactivator of Dnmt3a, resulted in inhibiting the promoter methylation of those four adipogenic TFs (Figure 4c). Taken together, these results suggest that the upregulated Dnmt3a functions for *de novo* methylation of key adipogenic TFs during the CI stage.

### Dnmt3a expression is upregulated by AP2*α* during the CI stage

**Figure 2:**
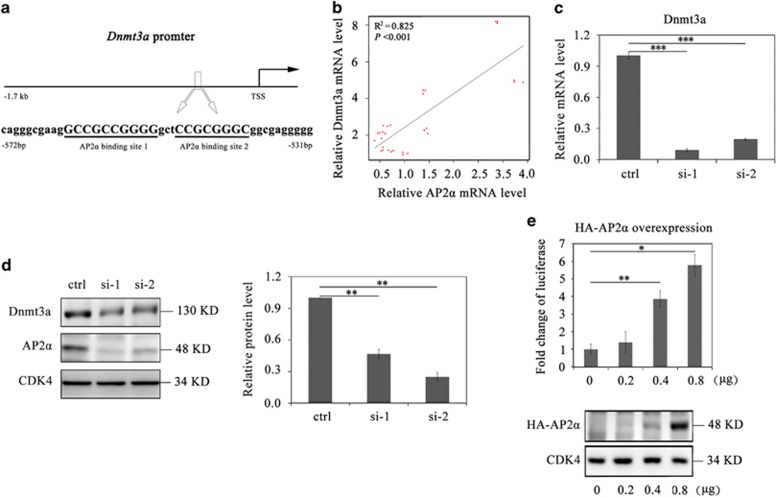
Dnmt3a is upregulated with increasing promoter methylation of adipogenic transcription factors during the stage of CI. (**a**) The mRNA expression of Dnmt3a during the process of 3T3-L1 preadipocyte differentiation. The mRNA level was determined by real-time PCR and normalized to glyceraldehyde-3-phosphate dehydrogenase mRNA. 'cyc' indicates the stage of cycling cells, 'ci' indicates the CI stage, 'mdi' indicates the stage of differentiation induction by hormone cocktail. Results were expressed as means±S.D. (*n*=3). Statistical significance is indicated: **P<0.01, ***P<0.001, NS=non-significant. (**b**) The protein expression of Dnmt3a was detected at indicated time points as described in (**a**) (left panel). Cyclin-dependent kinase 4 was used as the loading control. Relative quantification analysis of band densities was shown on the right panel. Data were presented as means±S.D. (*n*=3). Statistical significance is indicated: **P<0.01, ***P<0.001, NS=non-significant. (**c**) For promoter methylation analysis of adipogenic transcription factors, CpG island analysis of the 5' flanking and 5' untranslated sequences of murine *C/EBPβ*, *C/EBPα*, *PPARγ* and *Egr2* are shown. The red vertical line stands for a CpG site. The region indicated by arrows stands for the sequence for bisulfite sequencing. (**d**) Bisulfite sequencing results of murine *C/EBPβ*, *C/EBPα*, *PPARγ* and *Egr2* from 3T3-L1 preadipocytes under the indicated conditions. Each column stands for a CpG site. The methylation percentage of each CpG site was shown by blue in each column. And the non-methylation percentage of each CpG site was shown by yellow in each column. Data were presented as mean from three independent samples

We then tried to find out the possible regulator(s) for upregulating Dnmt3a during the CI stage in adipogenesis. On the basis of TRANSFAC Database, AP2*α* was predicted to be an upstream regulator for transactivating Dnmt3a, in which two reported AP2*α*-binding sites were found in the *Dnmt3a* proximal promoter region (Figure 2a).^22, 27, 28^ To verify our prediction, we first analyzed the expression profiles of AP2*α* at various time points of the whole 3T3-L1 differentiation process. Results from qRT-PCR and western blotting assays showed that the expression of AP2*α* both in mRNA and protein levels significantly increased during the CI stage and rapidly decreased upon the MDI induction (Supplementary Figures 2a and b), whereas an early adipogenic TF C/EBP*β* was upregulated within 2 days of the MDI induction and the late adipogenic TFs C/EBP*α* and PPAR*γ* were not upregulated until 4 days of the MDI induction (Supplementary Figures 2a and b). We further examined the expression profile of AP2*α* from cycling cells (cyc) to MDI-induced cells (mdi 48 h) in detail. The results showed that the expression of AP2*α* in both mRNA and protein levels began to increase at 12 h and reached a peak at 48 h of the CI stage, whereas the AP2*α* expression levels rapidly decreased upon the MDI induction (Supplementary Figures 2c and d). Taken together, these results indicate that the expression of AP2*α* is upregulated specifically during the CI stage of 3T3-L1 preadipocytes.

To investigate the subcellular localization of AP2*α* protein during the CI stage, 3T3-L1 preadipocytes were analyzed with nucleocytoplasmic separation assay and immunofluorescence staining at the cycling stage (cyc) and the CI stage (ci48 h), respectively. The western blotting results showed that AP2*α* proteins as well as nuclear Lamin B, in contrast to cytoplasmic tubulin, were detected only in the nuclear extracts at both cycling (cyc) and CI stages (ci48 h) (Supplementary Figure 2e). In agreement with western blotting results, immunofluorescence staining results displayed that increased AP2*α* proteins during the CI stage localized in the nuclei (Supplementary Figure 2f). Collectively, our data indicate that the induced AP2*α* proteins during the CI stage specifically localize in the nucleus, implying that AP2*α* might function as a transactivator during the CI stage.

As both the expression of AP2*α* and Dnmt3a specifically increased during the CI stage, we compared the mRNA expression profiles of these two genes with correlative coefficient analysis at indicated time points. The result showed a strong positive correlation between these two genes (Figure 2b; *R*^2^=0.825), implying that AP2*α* could be involved in the upregulation of Dnmt3a. To further test this hypothesis, we treated 3T3-L1 preadipocytes with AP2*α*-specific siRNAs (si-1 and si-2) at the beginning of CI (simply called ci0 h) and then detected the expression of Dnmt3a in these AP2*α* knockdown cells at 48 h of CI (simply called ci48 h). The efficient AP2*α* knockdown (Supplementary Figures 3a and b) resulted in markedly attenuated Dnmt3a in both mRNA (Figure 2c) and protein level (Figure 2d), supporting that AP2*α* participates in the upregulation of Dnmt3a during the CI stage. To address the AP2*α* function to transcriptionally regulate Dnmt3a, we co-transfected 3T3-L1 preadipocytes at ci0 h with a luciferase reporter plasmid containing the murine *Dnmt3a* promoter (ENSMUST00000020991, −1.7 to +0.1 kb) and a murine AP2*α* cDNA (NM_011547.4, pcDNA3.1-HA-AP2*α*) plasmid at various concentrations (Figure 2e, bottom panel), respectively. The cells were collected after 48 h of transfection and then subjected to luciferase analysis (see Materials and Methods). The results showed that overexpression of HA-AP2*α* resulted in the induced luciferase activities of *Dnmt3a* promoter in a dose-dependent manner (Figure 2e, top panel). Taken together, these data indicate that AP2*α* is upregulated specifically during the CI stage and functions for transcriptional activation of Dnmt3a expression.

### AP2*α* transcriptionally activates the *Dnmt3a* promoter by directly binding to a proximal promoter region

**Figure 3:**
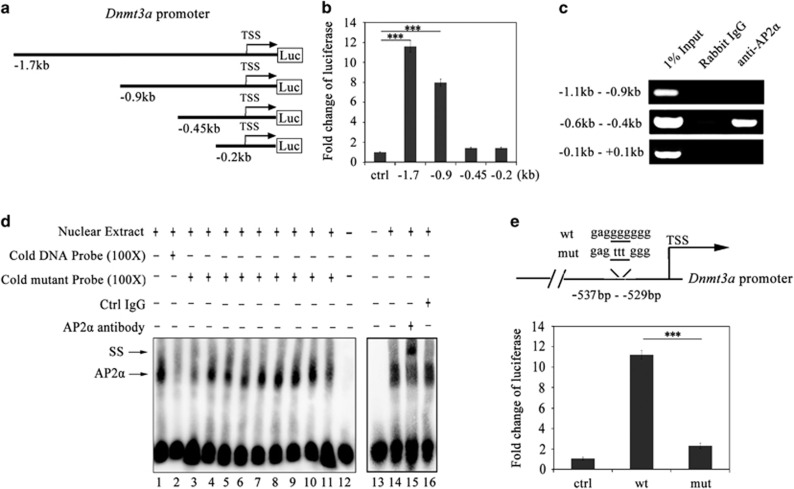
AP2*α* knockdown impairs both genome-wide DNA methylation and the promoter methylation of adipogenic TFs during the CI stage. (**a**) 5-methylcytosine immunofluorescence of 3T3-L1 preadipocytes at ci48 h. The cells were transiently transfected with either scramble siRNA (ctrl) or AP2*α* siRNAs (si-1 and si-2) at ci0 h and fixed at ci48 h for 5-methylcytosine immunofluorescence. Scale bars: 10 *μ*m. (**b**) Quantification analyses of total nuclear 5-methylcytosine densities in AP2*α* knockdown cells at ci48 h. Data were collected at the same voltage and were presented as mean±S.D. (*n*=3). Statistical significance is indicated: ***P<0.001. (**c**) DNA methylation status of *C/EBPβ*, *C/EBPα*, *PPARγ* and *Egr2* promoters. 3T3-L1 preadipocytes were transiently transfected with either scramble siRNA (ctrl) or AP2*α* siRNAs (si-1 and si-2) at ci0 h and collected at ci48 h. Genomic DNA samples were extracted and then subjected to bisulfite sequencing. Each column stands for a CpG site. The methylation percentage of each CpG site was shown by blue in each column. And the non-methylation percentage of each CpG site was shown by yellow in each column

To identify the *Dnmt3a* promoter region(s) responsible for the transactivation by AP2*α*, we generated a series of 5' deletion fragments of the *Dnmt3a* promoter inserted into the pGL3 basic luciferase vector (Figure 3a). And then, 3T3-L1 preadipocytes were transiently transfected with the luciferase reporters at ci0 h. After 48 h, the transfected cells were lysed and subjected to luciferase analysis. The results showed that the strong luciferase activities were detected when the 1.7 kb fragment (−1.7 to +0.1 kb) and the 0.9 kb fragment (−0.9 to +0.1 kb) of the *Dnmt3a* promoter were used, respectively, whereas the reporter activity was abolished when a fragment from −0.9 to −0.45 kb was deleted, indicating that this fragment was involved in AP2*α*-mediating transcription of *Dnmt3a* (Figure 3b).

Chromatin immunoprecipitation (ChIP) assay was further applied to investigate the association of endogenous AP2*α* with *Dnmt3a* promoter (see Materials and Methods). The results showed that the AP2*α* significantly bound to the fragment between −0.6 and −0.4 kb of the *Dnmt3a* promoter, whereas no obvious AP2*α* binding to the fragments between the −1.1 and −0.9 kb region or the −0.1 to +0.1 kb region of the *Dnmt3a* promoter was detected (Figure 3c), indicating that AP2*α* could only bind to the particular region of the *Dnmt3a* promoter. Further characterization of the AP2*α*-binding region between −0.6 and −0.4 kb of the *Dnmt3a* promoter was performed by using electrophoretic mobility shift assay (EMSA). Biotin-labeled DNA probes containing AP2*α* binding site 1 (wt1, −562 to −538 bp) and AP2*α* binding site 2 (wt2, −552 to −528 bp) were incubated with nuclear extracts from 3T3-L1 preadipocytes at ci48 h (Supplementary Figure 5a). The results showed that the AP2*α*-DNA complex formed when incubating nuclear extracts with wt2 probe (−552 to −528 bp) (Supplementary Figure 5a, lane 5; Supplementary Figure 5b, lane 2 and Figure 3d, lane 1), whereas the signal shift was competed away by the 100-fold excess of the cold wt2 probe (Supplementary Figure 5a, lane 6; Supplementary Figure 5b, lane 3 and Figure 3d, lane 2). In contrast, no AP2*α–*DNA complex formed when incubating nuclear extracts with wt1 probe (−562 to −538 bp) (Supplementary Figure 5a, lane 2). These results suggested that specific sequences in wt2 probe (−537 to –528 bp) were very important for AP2*α* binding. EMSA competition assay was then carried out with a series of cold mutant wt2 probes to find out the exact region for AP2*α* binding (Supplementary Figure 5b). The results showed that cold mutant wt2 probes with mutated AP2*α* binding site 2 sequence still had the competitive ability on binding the AP2*α*–DNA complex (Supplementary Figure 5b, lane 4–6), which indicated that the sequences in AP2*α* binding site 2 were not involved in AP2*α* binding. Interestingly, cold mutant wt2 probes with mutant sequence from −537 to −529 bp partially or greatly lost the competitive ability on binding the complex (Supplementary Figure 5b, lane 9–12 and Figure 3d, lane 3–11). In addition, the AP2*α–*DNA complex could be supershifted by the anti-AP2*α* antibody (Figure 3d, lane 15) and an anti-rabbit IgG antibody was used as negative control (Figure 3d, lane 16), confirming the specificity of the DNA sequences binding to AP2*α*. To verify whether the DNA sequences found by EMSA assay were responsible for *Dnmt3a* activation, we mutated the luciferase plasmid of *Dnmt3a* promoter (−1.7 to +0.1 kb) from −534 to −532 bp (Figure 3e). 3T3-L1 preadipocytes at ci0 h were transiently transfected with wild-type luciferase reporter plasmid (wt) and mutant luciferase reporter plasmid (mut), respectively. After 48 h, the transfected cells were lysed and subjected to luciferase analysis. The result showed that the mutant luciferase reporter plasmid significantly reduced the transcription activity induced by endogenous AP2*α* in 3T3-L1 preadipocytes (Figure 3e). Collectively, these results demonstrate that AP2*α* transcriptionally activates *Dnmt3a* through directly binding to a small proximal promoter region.

### AP2*α* is required for DNA methylation during the CI stage

**Figure 4:**
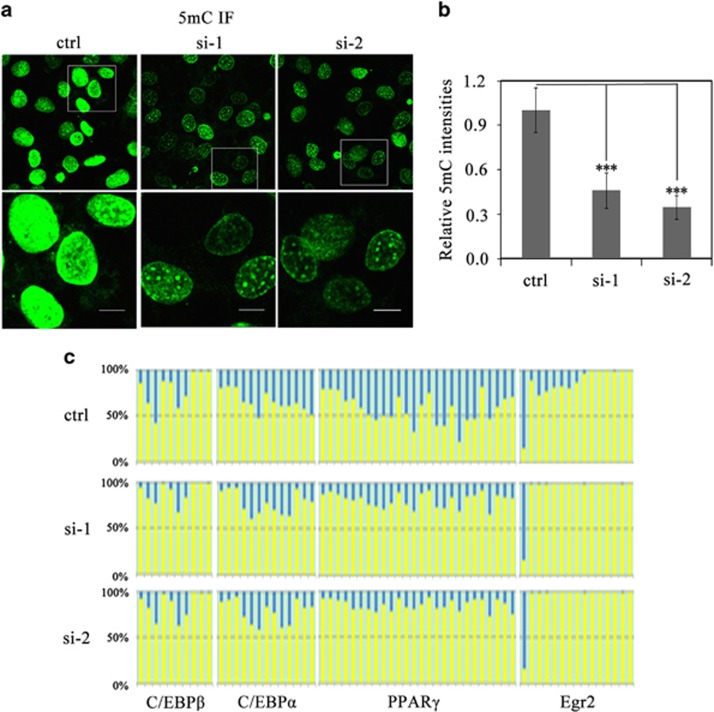
AP2*α* transactivates the *Dnmt3a* promoter by directly binding to a small proximal promoter region. (**a**) Schematic diagram of promoter segments of the murine *Dnmt3a* gene (−1.7 to +0.1 kb) inserted in the pGL3 basic luciferase vector. (**b**) A series promoter segments of the *Dnmt3a* gene were co-transfected with pRL-TK (Renilla) in 3T3-L1 preadipocytes at ci0 h. Results were expressed as firefly luciferase activity normalized to renilla luciferase activity. Error bars indicate S.D. (*n*=3). Statistical significance is indicated: ***P<0.001. (**c**) ChIP analysis of AP2*α* binding to the target region on the *Dnmt3a* promoter (−0.6 to −0.4 kb). Chromatin samples of 3T3-L1 preadipocytes at ci48 h were subjected to ChIP assays by using an AP2*α* antibody and normal rabbit IgG as a control. An upstream region (−1.1 to −0.9 kb) and a downstream region (−0.1 to +0.1 kb) in the *Dnmt3a* promoter were used as negative controls. (**d**) EMSA assay on the left panel (lane 1–12) showed the binding of endogenously expressed AP2*α* to the target region on the *Dnmt3a* promoter under the condition of biotin-labeled DNA probes and cold competitors. The right panel (lane 13–16) showed the supershift (SS) results of endogenously expressed AP2*α* in 3T3-L1 preadipocytes at ci48 h by anti-AP2*α* antibody. Addition of anti-AP2*α* antibody to the reaction resulted in the formation of the SS complex in lane 15. (**e**) Inactivation of the *Dnmt3a* promoter by mutating AP2*α* binding site in the *Dnmt3a* promoter. 3T3-L1 preadipocytes at ci0 h were co-transfected with a luciferase reporter plasmid of the *Dnmt3a* promoter (wt or mut) and pRL-TK. Results were expressed as firefly luciferase activity normalized to renilla luciferase activity. Error bars indicate S.D. (*n*=3). Statistical significance is indicated: ***P<0.001

We next sought to determine whether AP2*α* was involved in the regulation of DNA methylation during the CI stage. To investigate DNA methylation at the genomic level, we transiently transfected 3T3-L1 preadipocytes with AP2*α*-specific siRNAs (si-1 and si-2) at ci0 h and then subjected to 5-methylcytosine (5-MeC) immunofluorescence at ci48 h. Compared with the cells transfected with scramble siRNA (ctrl), the cells transfected with AP2*α*-specific siRNAs (si-1 and si-2) showed greatly reduced punctuated 5-MeC foci (Figure 4a). In addition, total nuclear 5-MeC intensities also decreased significantly when AP2*α* was knocked down (Figure 4b). These results indicate that AP2*α* knockdown impaired DNA methylation in the genomes of the preadipocytes during the CI stage. Furthermore, bisulfite sequencing was applied to investigate the promoter methylation status of *C/EBPβ*, *C/EBPα*, *PPARγ* and *Egr2* in AP2*α* knockdown cells, of which the promoter regions for bisulfite sequencing are shown in Figure 1c. The result showed the significant reduction in the promoter methylation of these four genes (Figure 4c). Taken together, our results indicate that AP2*α* participates in meditating DNA methylation during the CI stage.

### Impairment of adipogenesis by AP2*α* knockdown can be rescued by overexpression of Dnmt3a

**Figure 5:**
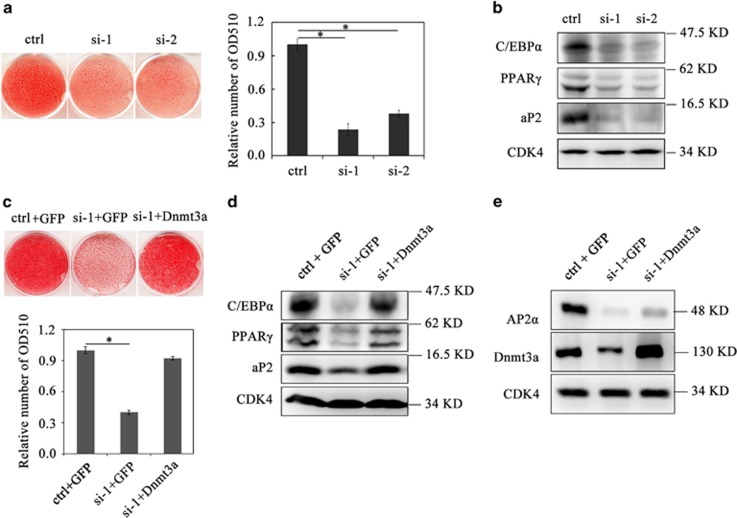
Overexpression of Dnmt3a rescues the impairment of adipogenesis induced by AP2*α* knockdown. **(a)** 3T3-L1 preadipocytes at ci0 h were transiently transfected with either scramble siRNA (ctrl) or AP2*α*-specific siRNAs (si-1 and si-2), and then induced with hormone cocktail at ci48 h. On day 8, the cells were stained with Oil-Red-O (left panel), and then extracted with isopropanol and measured at OD_510 nm_. The data were presented as means±S.D. (*n*=3) and shown on the right panel. Statistical significance is indicated: *P<0.01. (**b**) Western blotting analysis of adipogenic factors on the cells induced by MDI for 8 days. (**c**) For rescue study, 3T3-L1 cells at ci0 h were transiently co-transfected with three pairs of plasmids (scramble siRNA (ctrl) and GFP plasmid, AP2*α*-specific siRNA (si-1) and GFP plasmid, AP2*α*-specific siRNA (si-1) and Dnmt3a plasmid), respectively. The cells were then induced with hormone cocktail after 48 h transfection. On day 8 of MDI induction, the cells were stained with Oil-Red-O (top panel) and extracted with isopropanol and measured at OD_510 nm_ (bottom panel). The data were presented as means±S.D. (*n*=3). (**d**) Western blotting analysis of adipogenic factors on the cells induced by MDI for 8 days. (**e**) The expressions of Dnmt3a and AP2*α* were analyzed with western blotting. Cyclin-dependent kinase 4 served as the loading control

To test whether AP2*α* is required for adipogenesis, we knocked down AP2*α* in 3T3-L1 preadipocytes by transient transfection of siRNA at ci0 h, and then induced these transfected cells at ci48 h with MDI to differentiate. We found that adipogenesis was greatly impaired in the AP2*α* knockdown cells (si-1 and si-2) but not in cells treated with scramble siRNA (ctrl) (Figure 5a, left panel). This observation was confirmed by quantitative adipogenesis analysis for OD_510 nm_ (Figure 5a, right panel) and by western blotting of adipogenic protein markers (Figure 5b).

As AP2*α* was specifically upregulated during the CI stage and then quickly downregulated when the preadipocytes were induced by MDI (Supplementary Figures 2a–d), we propose that AP2*α* has its transactivating role only in the CI stage. To test this hypothesis, we treated the preadipocytes with AP2*α* siRNA (si-1) in three different periods (from ci0 h to ci48 h (ci0 h), ci48 h to mdi48 h (ci48 h) or mdi48 h to mdi 96 h (mdi48 h)), respectively. The results of Oil-Red-O staining showed that adipogenesis was greatly impaired when the cells were treated with AP2*α* siRNA (si-1) from ci0 h to ci48 h (Supplementary Figure 4a), whereas the treatments during the other two periods had no obvious effect (Supplementary Figure 4a). The results of quantitative analysis for OD_510 nm_ (Supplementary Figure 4b) and western blotting of adipogenic protein markers (Supplementary Figure 4c) were in consistent with the Oil-Red-O staining result. Taken together, these results illustrate that AP2*α* has its role in mediating adipogenesis mainly in the CI stage.

To test whether upregulation of Dnmt3a by AP2*α* during the CI stage is required for adipogenesis, we co-transfected 3T3-L1 preadipocytes with AP2*α*-specific siRNA (si-1) and a plasmid encoding Dnmt3a (NM_007872.4, pcDNA3.1-HA-Dnmt3a) at ci0 h simultaneously. For the control experiments, 3T3-L1 preadipocytes were co-transfected with scramble siRNA (ctrl) and a plasmid encoding GFP at ci0 h as positive control, and co-transfected with AP2*α*-specific siRNA (si-1) and a plasmid encoding GFP serving as negative control. After 48 h of transfection, the cells were subjected to MDI induction and then detected by Oil-Red-O staining on day 8. The result of Oil-Red-O staining and quantitative analysis for OD_510 nm_ showed that ectopic Dnmt3a overexpression in AP2*α* knockdown cells (Figure 5e) could greatly rescue the impairment of adipogenesis caused by AP2*α* knockdown (Figure 5c). Western blotting analysis showed that the expression of C/EBP*α*, PPAR*γ* and aP2 was recovered in these AP2*α* knockdown cells overexpressing ectopic Dnmt3a (Figure 5d). Collectively, these data conclude that AP2*α* functions on the upstream of Dnmt3a and has an essential role in granting preadipocyte the ability to differentiate through transactivating *Dnmt3a* expression during the CI stage.

## Discussion

Epigenetic modifications such as DNA methylation and histone modifications are involved in adipogenesis.^15, 29, 30, 31, 32, 33, 34, 35^ Our previous study has reasoned that the stage of CI might provide a special window for 3T3-L1 cells to establish an adipogenic competency program through epigenetic modifications. Dnmt3a has been implicated in epigenetic regulation in adipogenesis *in vitro* and *in* *vivo*.^15, 36^ In the present study, our data showed that Dnmt3a was upregulated in the CI stage in parallel with increasing promoter methylation of adipogenic genes. In addition, the DNA methylations at genome level of 3L3-L1 preadipocytes were also analyzed by whole-genome bisulfite sequencing assay and more than 23 000 DNA methylation sites were identified, in which about 23% DNA methylation sites were changed during the CI stage (data not shown). Taken together, the epigenetic programming of the preadipocyte genome is a major task to the licensing stage.

As the regulation of TFs is critical for adipocyte differentiation,^7, 8, 9, 10, 11^ the DNA methylation of TF promoters must be a major way for such transcriptional regulation.^15, 26, 37, 38, 39, 40, 41^ In agreement with this conclusion, increasing promoter methylation of adipogenic TFs catalyzed by Dnmt3a was observed (Figure 1d), whereas the downregulation of AP2*α*, which is the transactivator of Dnmt3a, resulted in the inhibition of DNA methylation of those TF promoters (Figure 4c). It is not fully clear why it would be advantageous to inhibit induction of adipogenic genes early in differentiation. In a study about Dnmt1 in adipogenesis, the researchers suggested that high methylation status of adipogenic genes might repress the transcriptional activities until the cells receive appropriate signals to differentiate.^42^ On the other hand, we also observed no changes of the promoter methylation status of several adipogenic TFs such as KLF4, KLF5 and GATA-2, implying that DNA methylation regulation might be selectively applied for particular TFs. In agreement with this observation, our genome-wide analysis showed that only about one-fourth of DNA methylation sites of the preadipocyte genomes were changed during the CI stage (data not shown).

So far, the studies on AP2*α* functions have been focused on its role as a repressor of the *C/EBPα* gene after MDI induction in the progress of adipogenesis.^22, 23, 24, 25, 26^ The present study indicates for the first time a role of AP2*α* in epigenetic programming during the CI stage as a positive regulator for transactivating Dnmt3a (Figures 2 and 3). It is interesting that AP2*α* might exert a biphasic regulatory role in adipogenesis, promoting adipogenesis during the CI stage while inhibiting differentiation at mitotic clonal expansion. As AP2*α* expression increased during the CI stage and then a rapid decline at 12 h upon MDI induction (Supplementary Figure 2), we don't think that AP2*α* could have its inhibitory role during mitotic clonal expansion under such condition. Further studies are warranted to explore the negative role of AP2*α* in the differentiation process of 3T3-L1 preadipocytes after the MDI induction.

In summary, we show here that Dnmt3a induced during the CI stage functions for regulating promoter methylation levels of critical adipogenic TFs. The upregulation of Dnmt3a expression is mediated by AP2*α*, which directly binds to its promoter region. The inhibition of AP2*α* during the CI stage resulted in impaired adipogenesis, whereas the overexpression of Dnmt3a could rescue the impairment of adipogenesis induced by knockdown of AP2*α* during the CI stage, supporting the existence of a functional link between these two genes.

## Materials and Methods

### 3T3-L1 cell culture, differentiation induction and Oil-red-O staining

3T3-L1 preadipocytes were cultured in Dulbecco's modified Eagle's medium supplemented with 10% calf serum (Invitrogen, Carlsbad, CA, USA). The 2-day post-confluent cells were induced for adipocyte differentiation following the protocol described previously.^15^ Cytoplasmic triglyceride droplets were visible by day 4. The differentiated 3T3-L1 adipocyte monolayer was stained with Oil-Red-O. The Oil-Red-O in triglyceride droplets was extracted with 100% isopropanol and OD_510 nm_ was determined.

### Plasmids constructs

To generate AP2*α* and Dnmt3a expression vector, cDNA isolated from 3T3-L1 cells was made by PCR with PCR Enhancer System (Invitrogen) and cloned into pcDNA3-HA (Invitrogen) between *Bam*HI and *Eco*RI. To generate Dnmt3a promoter construct for dual-luciferase assay, genomic DNA isolated from 3T3-L1 cells was made by PCR with PCR Enhancer System (Invitrogen) and cloned into pGL3 basic luciferase reporter expression vector (Promega, Madison, WI, USA) between *Kpn*I and *Sac*I. To generate Dnmt3a promoter segments for dual-luciferase assay, the indicated fragments were subcloned into pGL3 basic vector. To generate site-specific mutation of Dnmt3a promoter, mutation sites were introduced by PCR with QuikChange Site-Directed Mutagenesis Kit according to the manufacturer's protocols (Stratagene, Agilent Technologies, Inc., Santa Clara, CA, USA) and using the mutagenesis primers as following: forward, 5′-CCGCGGGCGGCGAG**TTT**GGGGGACGGGGGG-3′ reverse, 5′-CCCCCCGTCCCCC**AAA**CTCGCCGCCCGCGG-3′. All plasmids were confirmed by DNA sequencing.

### Quantitative real-time PCR

Total RNA extraction, first-strand cDNA synthesis and qRT-PCR were performed as we described previously.^15^ qRT-PCR assays using StepOnePlus Real-Time PCR system (Life Technologies Corporation, Carlsbad, CA, USA) were performed using SYBR Green quantitative PCR analysis reactions (TOYOBO, Osaka, Japan) under the following conditions: 10 min at 95 °C and then 40 cycles of 15 s at 94 °C, 30 s at 62 °C and 20 s at 72 °C. Each sample was assayed in triplicate. Glyceraldehyde-3-phosphate dehydrogenase was used as an endogenous reference to assess the relative level of mRNA transcript. The primers used for PCR analysis were listed in Supplementary Materials and Methods.

### Western blotting assay

For cytoplasmic and nuclear extracts preparation, 3T3-L1 preadipocytes at ci48 h were treated by NE-PER Nuclear and Cytoplasmic Extraction Reagents (Thermo Fisher Scientific Inc., Rockford, IL, USA). The procedure was carried out according to the manufacturer's instructions. After separation, the components were prepared with 5 × SDS lysis buffer. For preparation of the whole-cell extracts, cells were rinsed with PBS and harvested with 1 × SDS lysis buffer. Protein content was quantified by a Lowry assay. Proteins were separated by sodium dodecyl sulfate-polyacrylamide gel electrophoresis and transferred to PVDF membranes. Following transfer, the membrane was blocked in 5% BSA for 1h at room temperature and probed with antibody. The following primary antibodies were used: anti-AP2*α* (#3215; Cell signaling, Boston, MA, USA), anti-Dnmt3a (#2160; Cell signaling), anti-CDK4 (sc-260, Santa Cruz, CA, USA), anti-C/EBP*β* (sc-150; Santa Cruz), anti-C/EBP*α* (sc-61; Santa Cruz), anti-PPAR*γ* (sc-7273; Santa Cruz), anti-Lamin B (sc-6216; Santa Cruz), anti-rabbit IgG (sc-2027; Santa Cruz), anti-aP2 (AF1443; R & D Systems, Minneapolis, MN, USA), anti-Tubulin (T6199; Sigma, St. Louis, MO, USA), anti-HA (Tiangen, AB104) and anti -5-MeC (ab10805; Abcam, Cambridge, MA, USA). The Immobilon Western Chemiluminescence HRP Substrate was from Millipore Corporation (Billerica, MA, USA).

### Immunostaining for AP2*α* and 5-methylcytosine

3T3-L1 preadipocytes grown on coverslips were fixed in 4% paraformaldehyde for 15 min and permeabilized for 30 min with cold PBS containing 1% Triton X-100 in room temperature. For AP2*α* staining, cells were blocked for 1h with PBS, 5% BSA and 0.1% Tween at room temperature followed by AP2*α* antibody (1:200) that was carried out in the same buffer at 4°C  overnight. The coverslips were subsequently washed three times in PBS, 0.25% BSA and 0.1% Tween-20 (5 min each wash) and incubated with Alexa fluorophore conjugated secondary antibody (Molecular Probes, Carlsbad, CA, USA) for 1h at room temperature in the dark. Finally, the coverslips were covered with anti-fade mounting solution with 4′,6′-diamidino-2-phenylindole (Invitrogen). For 5-MeC staining, cells were treated in 70% formamide, 2±SSC for 5 min in 85°C after permeabilization with Triton X-100. And then cells were dehydrated in ice-cold ethanol. After brief air drying, the coverslips were blocked and incubated with 5-MeC antibody (1:50) for 4h at room temperature, followed by washing with PBST (0.05% Tween-20) three times and incubation with FITC-conjugated anti-sheep secondary antibody (1:800) at room temperature for 1 h. Observations were performed with a Leica TCS SP2+ABOS confocal fluorescence microscope system (Leica Microsystems, Wetzlar, Germany) using a HC±PL Apo±63 oil immersion objective (NA=1.4) and an excitation wavelength of 488 nm. For 5-MeC staining, the same gain, black-level and aperture parameters were used. Nuclear densities were measured by manual outlining with Leica confocal analysis software (Leica Microsystems, Wetzlar, Germany).

### AP2*α* knockdown and Dnmt3a overexpression

For AP2*α* knockdown, cycling 3T3-L1 preadipocytes were transfected with 100 nM chemically synthesized siRNA using Lipofectamine 2000 (Invitrogen). Two commercial pre-designed siRNAs for AP2*α* were used (Invitrogen, s74845 and s74846). A scramble sequence (5′-UUCUCCGAACGUGUCACGUTT-3′) was synthesized as an internal control. The efficiency of siRNA knockdown was confirmed by western blotting assay. Chemically synthesized scramble siRNA was made by Gene Pharma Corporation (Shanghai, China).

For Dnmt3a overexpression in AP2*α* knockdown cells, 3T3-L1 preadipocytes were plated for 24 h and transiently transfected with AP2*α* siRNA (s74845) combined with Dnmt3a expression plasmid using Lipofectamine 3000 (Invitrogen). The amounts of DNA, RNA and Lipofectamine 3000 used for 6-well plates are 2 *μ*g, 50 pmol and 7.5 *μ*l. A scramble siRNA combined with GFP expression plasmid was used as controls. After 60 h transfection, cells were harvested for western blotting assay or induced to differentiation.

### Dual-luciferase assay

3T3-L1 cells were seeded into a 24-well plate and transfected with the luciferase reporter plasmids as indicated using Lipofectamine 2000 (Invitrogen). pRL-TK vector that carries renilla luciferase was also transfected as an internal control for transfection efficiency. The transfected cells were lysed after 48 h and subjected to analysis of firefly and renilla luciferases with the Dual Luciferase Reporter Assay System (Promega). The firefly luciferase values were normalized to Renilla values.

### Chromatin immunoprecipitation assay

ChIP experiment was performed using a commercial kit (Millipore) according to the manufacturer's instructions. Briefly, 2-day post-confluent cells were cross-linked with 1% formaldehyde for 10 min at 37 °C, followed by cell lysis and sonication. Then proteins cross-linked with DNA were immunoprecipitated with 1 *μ*g of anti-AP2*α* antibody or anti-rabbit IgG and 60 *μ*l of salmon sperm DNA/protein-A-agarose beads. The protein-A-agarose antibody–protein complexes were washed extensively and eluted, according to the manufacturer's recommendations. The cross-link was reversed and proteins were digested by proteinase-K for 1 h at 45 °C. DNA was recovered by phenol/chloroform extraction and ethanol precipitation, and used as template for PCR. The sequences of primers for PCR were listed in Supplementary Materials and Methods.

### Electrophoretic mobility shift assay

The nuclear extracts of 3T3-L1 preadipocytes at ci48 h were prepared as we previously described.^15^ The sequence of the oligonucleotide DNAs containing the exact binding sequences of AP2*α* in the *Dnmt3a* promoter is 5′-GGGCTCCGCGGGCGGCGAGGGGGGGG-3′. Other DNA sequences used for EMSA are listed in Supplementary Materials and Methods. The double-strand complementary oligonucleotide DNAs were annealed and end labeled using a Biotin 3' End DNA Labeling kit (Pierce Biotechnology, Fremont, CA, USA), and EMSAs were performed using a LightShift Chemiluminescent EMSA kit according to the company's instruction (Pierce Biotechnology).

### Bisulfite sequencing

Genomic DNA from 3T3-L1 preadipocytes at indicated times was prepared by phenol/chloroform extractions. Bisulfite conversion was performed using EpiTech Bisulfite kit (Qiagen, Hilden, Germany). The converted DNA was used as template to amplify DNA sequence covering the putative CpG sites at adipogenic genes' promoter regions shown in Figure 2. Each pair of the amplification primers was end-modified to add three adaptors for three independent samples from one time point. Amplification was done for library construction and next-generation bisulfite sequencing was done to obtain raw data. The detailed sequences for primers were described in Supplementary Materials and Methods.

### Statistics

All data are expressed as mean±S.D. For statistical analysis, the *P-*value was calculated using a two-tailed unpaired Student's *t*-test with *P*<0.05 being considered statistically significant.
