# Supplementary material for: Epigenetic programming of Dnmt3a mediated by AP2α is required for granting preadipocyte the ability to differentiate
Source: Cell Death Dis. 2016 Dec 1;7(12):e2496–. doi: 10.1038/cddis.2016.378 (PMC5261006; doi:10.1038/cddis.2016.378)
Supplement: Supplementary Figures [file cddis2016378x1.docx]

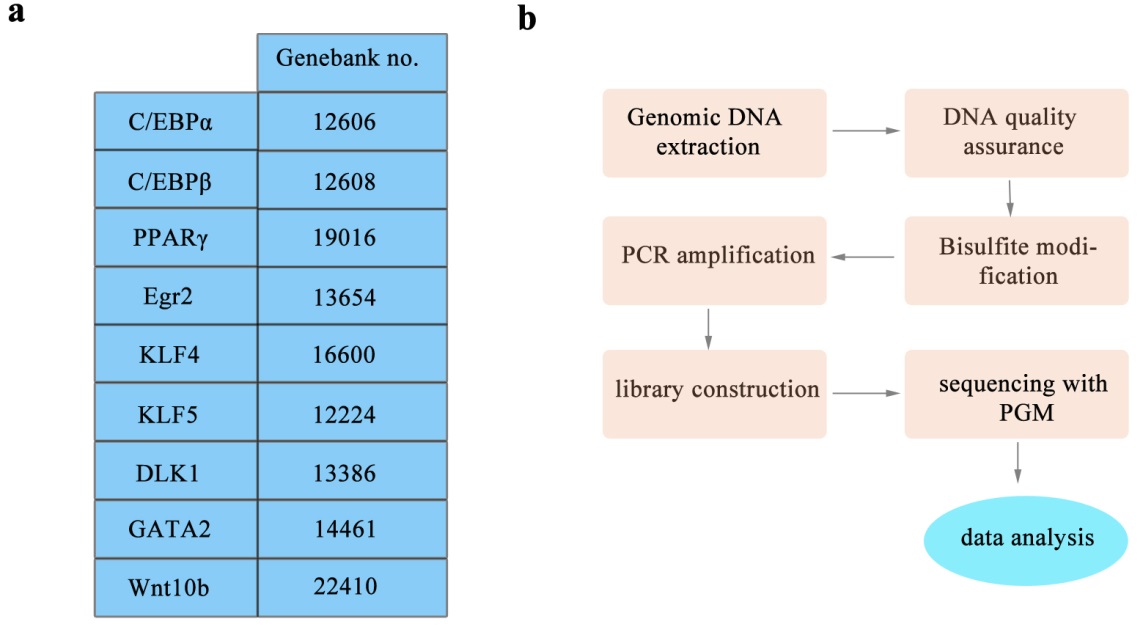


**Supplementary Figure 1** Experiment procedure for bisulfite sequencing. (**a**) 9 key adipogenic transcription factors were chosen for bisulfite sequencing. (**b**) Schematic diagram of experiment procedure for bisulfite sequencing.


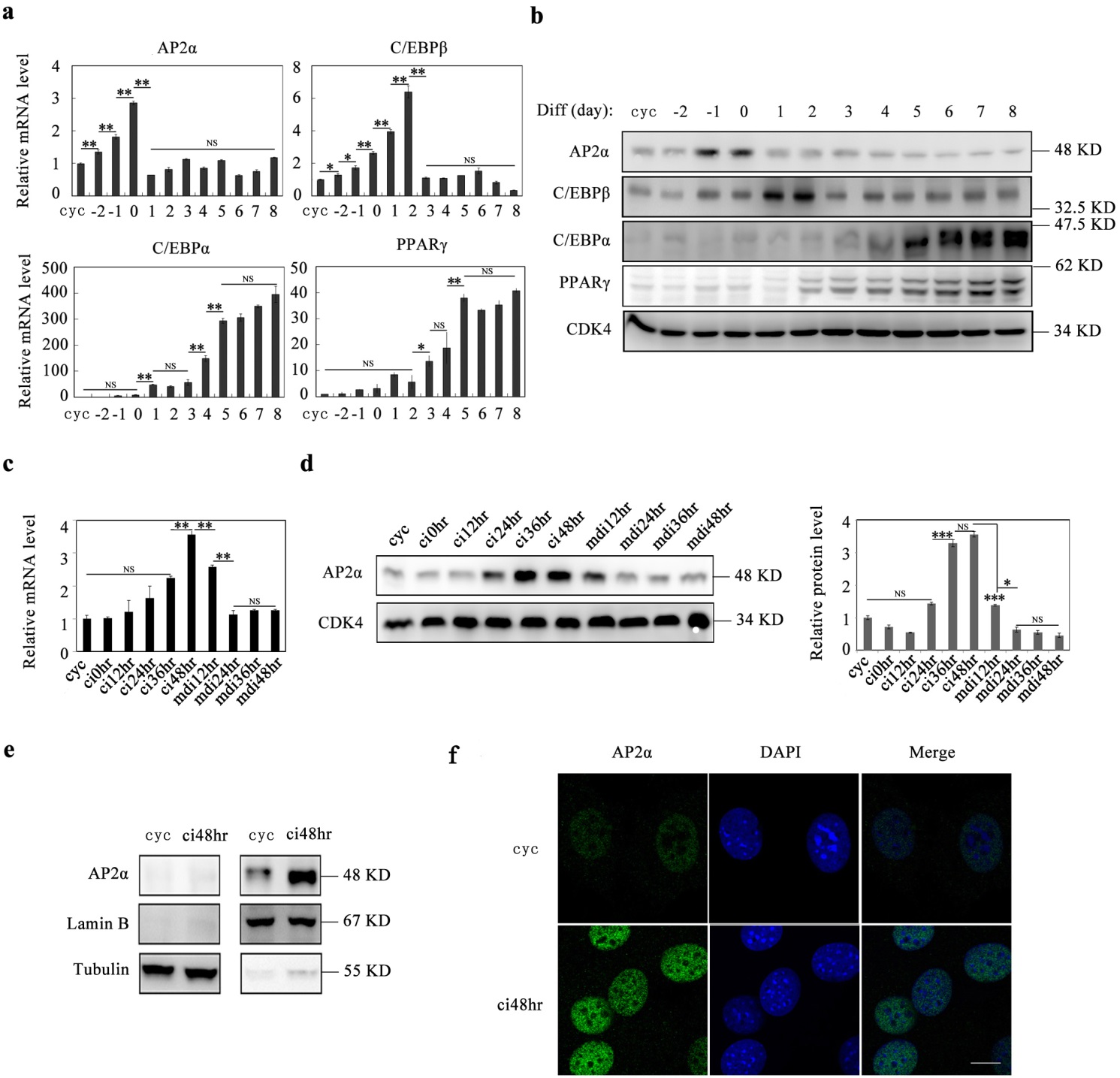


**Supplementary Figure 2** AP2α is elevated during the CI-stage in 3T3-L1 preadipocyte differentiation. (**a**) The mRNA expressions of AP2α, C/EBPβ, C/EBPα and PPARγ during the time course of 3T3-L1 preadipocyte differentiation. The mRNA level was determined by real-time PCR and normalized to GAPDH mRNA. The numbers indicate the day(s) induced by hormone-cocktail. Results were expressed as means ± S.D. (n=3). Statistical significance is indicated: **P* < 0.05, ***P* < 0.01, NS=non-significant. (**b**) Detection of protein expressions of AP2α, C/EBPβ, C/EBPα and PPARγ by western blotting at indicated time-points as described in (**a**). CDK4 was used as the loading control. (**c**) The mRNA expression of AP2α was detected by real-time PCR and normalized to GAPDH mRNA at indicated time-points of the CI-stage (ci0hr to ci48hr) and the MDI-induced stage (mdi12hr to mdi48hr). Data were presented as means ± S.D. (n=3). Statistical significance is indicated: ***P* < 0.01, NS=non-significant. (**d**) The protein expression of AP2α was detected by western blotting (left panel) at indicated time-points as described in (**c**), and the quantified results was shown on the right panel. CDK4 was used as the loading control. Data were presented as means ± S.D. (n=3). Statistical significance is indicated: **P* < 0.05, ****P* < 0.001, NS=non-significant. (**e**) The levels of AP2α in cytoplasmic and nuclear extracts were determined by western blotting at the indicated time-points. Lamin B served as a nuclear marker and tubulin served as a cytoplasmic marker. (**f**) Localization of AP2α was detected by immunofluorescence at the indicated time-points. Representative fluorescence images from cyc and ci48hr preadipocytes were stained with anti-AP2α antibody (green) and DAPI (blue). Scales bar, 10μm.


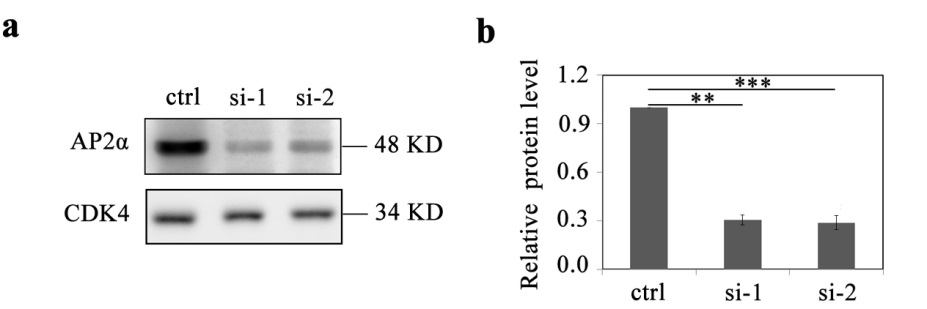


**Supplementary Figure 3** Knockdown effect of AP2α-specific siRNAs. (**a**) To detect the knockdown effect with AP2α-specific siRNAs, 3T3-L1 preadipocytes at ci0hr were transiently transfected with either scramble siRNA (ctrl) or AP2α-specific siRNAs (si-1 and si-2). Whole cell extracts were collected at ci48hr and subjected to western blotting. CDK4 served as the loading control. (**b**) Relative quantification analyses of AP2α protein level were shown. Data were presented as means ± S.D. (n=3). Statistical significance is indicated: ***P* < 0.01, ****P* < 0.001.


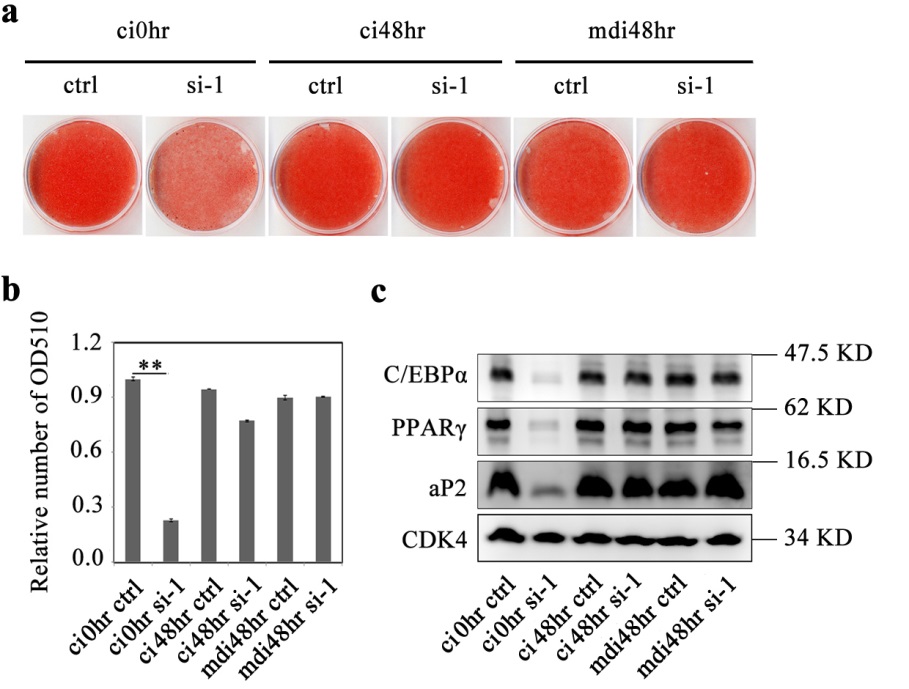


**Supplementary Figure 4** AP2α plays an important role during the CI-stage in 3T3-L1 preadipocyte differentiation. (**a**) 3T3-L1 cells were transiently transfected with either scramble siRNA (ctrl) or AP2α-specific siRNA (si-1) at the indicated time-points described as Fig. 1a legend, and then induced by the hormone-cocktail. On day 8 of the MDI-induction, the cells were stained with Oil-Red-O. (**b**) The Oil-Red-O staining cells were extracted with isopropanol and quantified by measuring the OD_510nm_. The data were presented as means ± S.D. (n=3). Statistical significance is indicated: ***P* < 0.01. (**c**) Western blotting analysis of C/EBPα, PPARγ and aP2. Whole cell extracts on day 8 of the MDI-induction were collected and then subjected to western blotting. CDK4 served as the loading control.


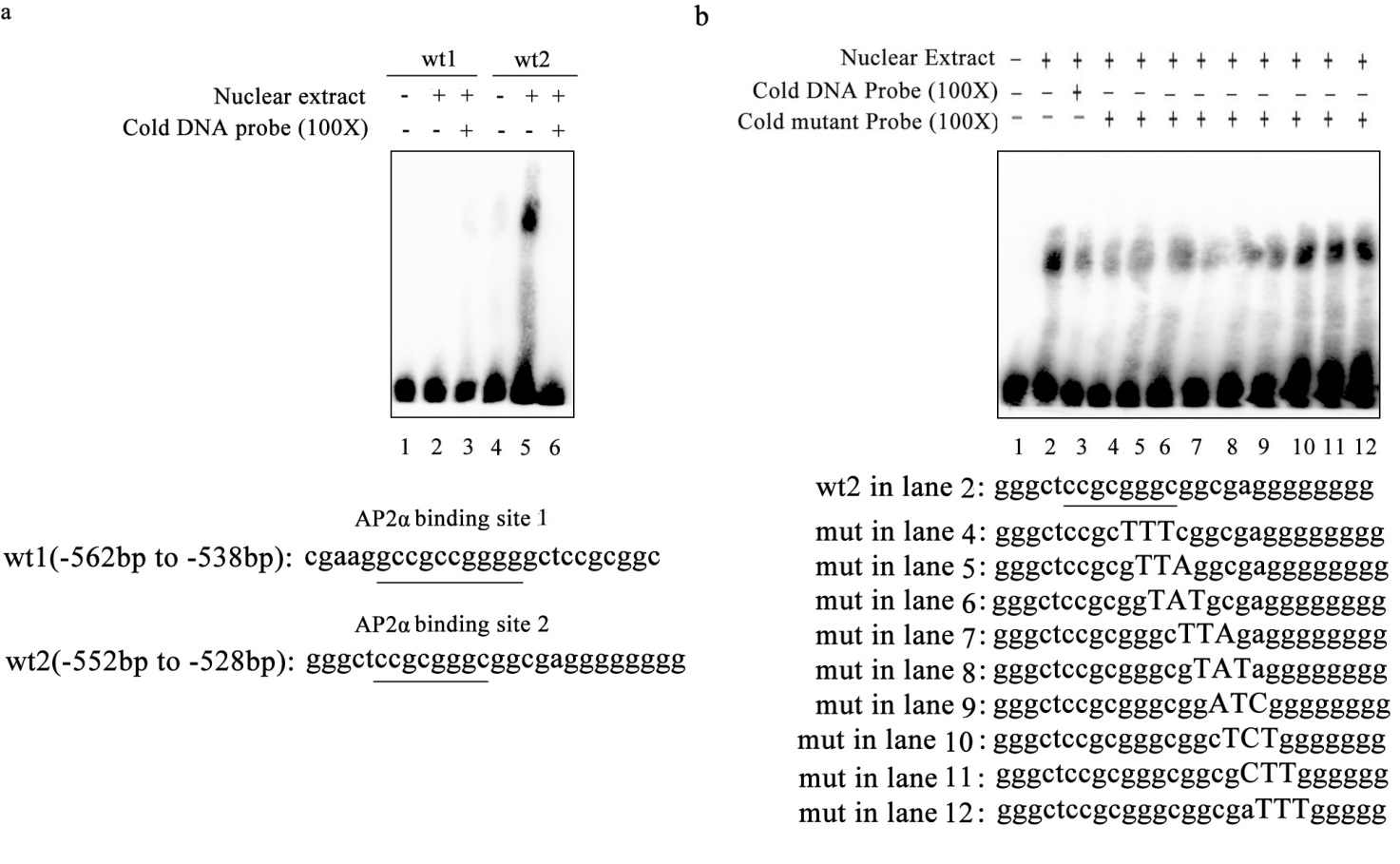


**Supplementary Figure 5** (**a**) EMSA assay demonstrates that the AP2α-DNA complex formed *in vitro* by 3T3-L1 preadipocytes nuclear extracts with wt2 probes could be diminished by excess cold wt2 probes (lane 5 and 6). The nuclear extracts from post-confluent cells were prepared for EMSA assay. The wt1 probes (-562 bp to -538 bp) and wt2 probes (-552 bp to -528 bp), respectively, contain AP2α binding site 1 and AP2α binding site 2. (**b**) EMSA assay with mutant cold competitors was used to test the exact region for AP2α binding. Biotin-labelled wt2 probes were incubated with the nuclear extracts from post-confluent 3T3-L1 cells. Wild-type cold wt2 competitors (lane 3) or mutant cold wt2 competitors (lane 4 to lane 12) were added to the reactions respectively.
