# Supplementary material for: Epigenetic programming of Dnmt3a mediated by AP2α is required for granting preadipocyte the ability to differentiate
Source: Cell Death Dis. 2016 Dec 1;7(12):e2496–. doi: 10.1038/cddis.2016.378 (PMC5261006; doi:10.1038/cddis.2016.378)
Supplement: Supplementary methods [file cddis2016378x2.docx]

**Supplementary Methods**

**Primers for qRT-PCR.** Primers used in qRT-PCR analysis were listed as following: AP2α (forward, 5’-GGAGACGTAAAGCTGCCAAC-3’; reverse, 5’-TTCTTGCCA

CTTGCTCATTG-3’), C/EBPβ (forward, 5’-ACCGGGTTTCGGGACTT

GA-3’; reverse, 5’-GTTTCGATATCACTGGAG-3’), C/EBPα (forward, 5’-CAGGGC

AGGAGGAAGATACA-3’; reverse, 5’-GGAAACCTGGCCTGTTGTAA-3’), PPARγ (forward, 5’-TTTTCAAGGGTGCCAGTTTC-3’; reverse, 5’-AATCCTTGGCCCTC

TGAGAT-3’), aP2 (forward, 5’-TCACCTGGAAGACAGCTCCT-3’; reverse, 5’-AAT

CCCCATTTACGCTGATG-3’), Dnmt3a (forward, 5’-ACCGTGTCTCTGCCATACC

T-3’; reverse, 5’-AAAGTGCCCATGAGAAAACC-3’), GAPDH (forward, 5’-TGGC

AAAGTGGAGATTGTTGCC-3’; reverse, 5’-AAGATGGTGATGGGCTTCCCG-3’).

**Primers for ChIP assay.** Primer pair designed for -1.1 kb - -0.9 kb region was forward primer 5’-AGCATTGAGGGTCTGCAAAT-3’ and reverse primer 5’-ACTGGAGCTTGGCACTGAAT-3’. Primer pair designed for -0.6 kb - -0.4 kb region was forward primer 5’-GTGGAGGTCGGGAGAACTG-3’ and reverse primer 5’-CATCCAGCACTGGTCGTAGG-3’. Primer pair designed for -0.1 kb - +0.1 kb region was forward primer 5’-GAAGGAGGAAGGCGGAGAGAAG-3’ and reverse primer 5’-CAGGCCGTGCTGGTTACCGTGT-3’.

**Competitors used for EMSA assay.** DNA sequences for wild-type competitor and a series of mutant competitors were listed as following: wild-type competitor forward 5’-GGGCTCCGCGGGCGGCGAGGGGGGGG-3’; mutant competitor (used in lane 3) forward 5’-GGGCTCCGCGGGCGG**ATC**GGGGGGGG-3’; mutant competitor (used in lane 4) forward 5’-GGGCTCCGCGGGCGGC**TCT**GGGGGGG-3’; mutant competitor (used in lane 5) forward 5’-GGGCTCCGCGGGCGGCG**CTT**GGGGG

G-3’; mutant competitor (used in lane 6) forward 5’-GGGCTCCGCGGGCGGCGA

**TTT**GGGGG-3’; mutant competitor (used in lane 7) forward 5’-GGGCTCCGCGGG

CGGCGAG**TTT**GGGG-3’; mutant competitor (used in lane 8) forward 5’-GGGCTC

CGCGGGCGGCGAGG**TTT**GGG-3’; mutant competitor (used in lane 9) forward 5’-GGGCTCCGCGGGCGGCGAGGG**TTT**GG-3’; mutant competitor (used in lane 10) forward 5’-GGGCTCCGCGGGCGGCGAGGGG**TTT**G-3’; mutant competitor (used in lane 11) forward 5’- GGGCTCCGCGGGCGGCGAGGGGG**TTT**-3’.

**Primers for bisulfite sequencing are listed as following:**

| Cebpa_322AF: | ATCACGGAAAGAGAGGTGTTTTGTTTGGA |
| --- | --- |
| Cebpa_322AR: | ATCACGACTTCCAACCAACACTAAAAAACC |
| Cebpb_305AF: | ATCACGTTGTTTTTTAAGAGTTGGGGGTT |
| Cebpb_305AR: | ATCACGTCACRCTAAAACCCCTCCC |
| DLK1_249AF: | ATCACGTGTGTTTGTTGGGTGATTTTATAA |
| DLK1_249AR: | ATCACGCTCCTACCTATACTACCCCTCCAC |
| Egr2_270AF: | ATCACGTATATATGGATTGAGGAATAGGGT |
| Egr2_270AR: | ATCACGCCAAAAAACAAAACTACCAAC |
| GATA2_285AF: | ATCACGGTTAGTTGGATTTGGGTTGGT |
| GATA2_285AR: | ATCACGTAAACTACCTAACCCCCTACCAAC |
| KLF4_244AF: | ATCACGTGGTAGGATTTTYGAGTTTAG |
| KLF4_244AR: | ATCACGTACCATAATAACTAAATAAACAAACTC |
| KLF5_272AF: | ATCACGAATTTGTTAGAGAAGTTGTGTATAAATTG |
| KLF5_272AR: | ATCACGAACTCCACCAACAACCTAAAA |
| PPAR_333AF: | ATCACGGTGAGGAGTAAGGYGGTTAGGTAA |
| PPAR_333AR: | ATCACGCAACRCCCCAAATCTCTTCT |
| Wnt10b_215AF: | ATCACGTGAAAGATTTTGTTATTGGGATTAGA |
| Wnt10b_215AR: | ATCACGCCACCCCCTAACTTAACCAA |
|  |  |
| Cebpa_322BF: | CGATGTGAAAGAGAGGTGTTTTGTTTGGA |
| Cebpa_322BR: | CGATGTACTTCCAACCAACACTAAAAAACC |
| Cebpb_305BF: | CGATGTTTGTTTTTTAAGAGTTGGGGGTT |
| Cebpb_305BR: | CGATGTTCACRCTAAAACCCCTCCC |
| DLK1_249BF: | CGATGTTGTGTTTGTTGGGTGATTTTATAA |
| DLK1_249BR: | CGATGTCTCCTACCTATACTACCCCTCCAC |
| Egr2_270BF: | CGATGTTATATATGGATTGAGGAATAGGGT |
| Egr2_270BR: | CGATGTCCAAAAAACAAAACTACCAAC |
| GATA2_285BF: | CGATGTGTTAGTTGGATTTGGGTTGGT |
| GATA2_285BR: | CGATGTTAAACTACCTAACCCCCTACCAAC |
| KLF4_244BF: | CGATGTTGGTAGGATTTTYGAGTTTAG |
| KLF4_244BR: | CGATGTTACCATAATAACTAAATAAACAAACTC |
| KLF5_272BF: | CGATGTAATTTGTTAGAGAAGTTGTGTATAAATTG |
| KLF5_272BR: | CGATGTAACTCCACCAACAACCTAAAA |
| PPAR_333BF: | CGATGTGTGAGGAGTAAGGYGGTTAGGTAA |
| PPAR_333BR: | CGATGTCAACRCCCCAAATCTCTTCT |
| Wnt10b_215BF: | CGATGTTGAAAGATTTTGTTATTGGGATTAGA |
| Wnt10b_215BR: | CGATGTCCACCCCCTAACTTAACCAA |
|  |  |
| Cebpa_322CF: | TTAGGCGAAAGAGAGGTGTTTTGTTTGGA |
| Cebpa_322CR: | TTAGGCACTTCCAACCAACACTAAAAAACC |
| Cebpb_305CF: | TTAGGCTTGTTTTTTAAGAGTTGGGGGTT |
| Cebpb_305CR: | TTAGGCTCACRCTAAAACCCCTCCC |
| DLK1_249CF: | TTAGGCTGTGTTTGTTGGGTGATTTTATAA |
| DLK1_249CR: | TTAGGCCTCCTACCTATACTACCCCTCCAC |
| Egr2_270CF: | TTAGGCTATATATGGATTGAGGAATAGGGT |
| Egr2_270CR: | TTAGGCCCAAAAAACAAAACTACCAAC |
| GATA2_285CF: | TTAGGCGTTAGTTGGATTTGGGTTGGT |
| GATA2_285CR: | TTAGGCTAAACTACCTAACCCCCTACCAAC |
| KLF4_244CF: | TTAGGCTGGTAGGATTTTYGAGTTTAG |
| KLF4_244CR: | TTAGGCTACCATAATAACTAAATAAACAAACTC |
| KLF5_272CF: | TTAGGCAATTTGTTAGAGAAGTTGTGTATAAATTG |
| KLF5_272CR: | TTAGGCAACTCCACCAACAACCTAAAA |
| PPAR_333CF: | TTAGGCGTGAGGAGTAAGGYGGTTAGGTAA |
| PPAR_333CR: | TTAGGCCAACRCCCCAAATCTCTTCT |
| Wnt10b_215CF: | TTAGGCTGAAAGATTTTGTTATTGGGATTAGA |
| Wnt10b_215CR: | TTAGGCCCACCCCCTAACTTAACCAA |
